# A Three-Step Submandibular Retropharyngeal Approach to the Craniovertebral Junction: Is Less Always More?

**DOI:** 10.3390/jcm13133755

**Published:** 2024-06-27

**Authors:** Massimiliano Visocchi, Alberto Benato, Mario Flavio Davila, Ali Abdelnabi Bayati, Fabio Zeoli, Francesco Signorelli

**Affiliations:** 1 Operative Unit and Academic Research Center on Surgical Approaches to the Craniovertebral Junction, Università Cattolica del Sacro Cuore, 00168 Rome, Italy; 2 Department of Neurosurgery, Fondazione Policlinico Universitario “A. Gemelli” IRCCS, 00168 Rome, Italy; 3 Guatemalan Institute of Social Security, 01003 Ciudad de Guatemala, Guatemala; 4 Shmaisani Hospital, Amman, Jordan

**Keywords:** craniovertebral junction, submandibular approach, chordoma, retropharyngeal, endoscope-assisted surgery

## Abstract

**Background**: Accessing the craniovertebral junction poses unique challenges due to its anatomical complexity and proximity to critical structures, such as the cord–brainstem junction, great vessels of the neck, cranial nerves, oropharynx, and rhinopharynx. Among the approaches that have been developed over the years, the submandibular retropharyngeal approach offers good antero-lateral access without the need of transgressing mucosal layers. In its traditional form, however, this approach involves multiple sequential steps and requires intricate dissection, extensive retraction, and meticulous maneuvering, which can increase operative time and produce approach-related morbidity. **Methods**: With this paper, we propose a simplified technique for a submandibular retropharyngeal approach involving only three surgical steps. The advantages and limitations of this technique are illustrated through three surgical cases of neoplastic and degenerative craniovertebral junction pathologies. **Results**: In two out of the three cases, our technique allowed for a wide exposure of the lesions that could be resected totally or sub-totally with good outcome. In one case with involvement of the clivus and the occipital condyle, the exposure was inadequate; a biopsy was obtained, and the lesion was subsequently resected via and endoscopic transmucosal approach. **Conclusions**: Our technique represents a significant simplification of the traditional submandibular retropharyngeal approach; with appropriate indication, it permits a fast, safe, and adequate exposure of craniovertebral junction pathologies.

## 1. Introduction

The submandibular retropharyngeal approach (SRA), also referred to as the anterolateral high cervical approach [1,2,3,4,5], exposes the area above the hyoid bone and medial to the neurovascular bundle of the neck in the parapharyngeal space. This approach can provide a wide anterior exposure of the craniovertebral junction (CVJ) without the need to cut through mucosal layers; however, several risks are potentially associated with SRA, such as injury to the hypoglossal and superior laryngeal nerves, as well as injury to the marginal mandibular branch of the facial nerve either directly or secondarily to vigorous retraction during surgery.

The traditional description of the approach by McAfee and colleagues [1] describes several steps (namely, 11 steps), from patient positioning to final exposure of the prevertebral fascia with retraction of pharyngeal structures [6]. After the paper of De Bonis et al., who proposed a four-step modification of the previous McAfee approach [6], we propose a three-step SRA that allows for obtaining more straightforward access to CVJ pathologies, minimizing complications. Such an approach might replace the classic anterior transmucosal approaches to avoid their dreaded complications [7].

## 2. Materials and Methods

We describe our surgical technique and illustrate its application in 3 cases of neoplastic pathologies of the CVJ.

**Figure 1:**
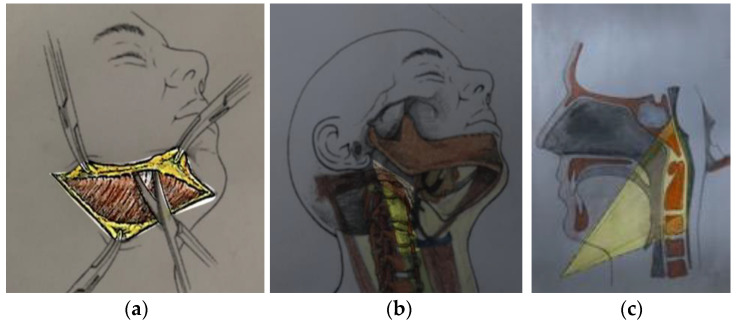
(**a**–**c**) Drawings illustrating the skin incision, anatomical corridor, and obtainable exposure of the traditional submandibular approach as described by Salle and colleagues [8].

Our technique involves three steps:

Step 1: head positioning in the traditional way, as described in the literature (hyperextension and contralateral rotation of 30°).
Step 2: a horizontal incision of the skin and platysma is performed from the midline to lateral aspect of the mandible, two fingers’ breadth below the bone (to avoid damage to the submandibular gland and branches of the facial nerve) (Figure 1).
Step 3: direct exposure of the retropharyngeal space by finger dissection starting by palpating the greater horn of the hyoid bone (infero-medially) and then following the natural anatomical plane between the medial cervical compartment (bounded by the buccopharyngeal fascia and comprising the laryngeal and pharyngeal structures) and the carotid sheath. The retropharyngeal space is composed of loose fibro-adipose tissue connecting the prevertebral fascia (posteriorly) to the pharyngeal muscles enveloped by the buccopharyngeal fascia (anteriorly).

**Figure 2:**
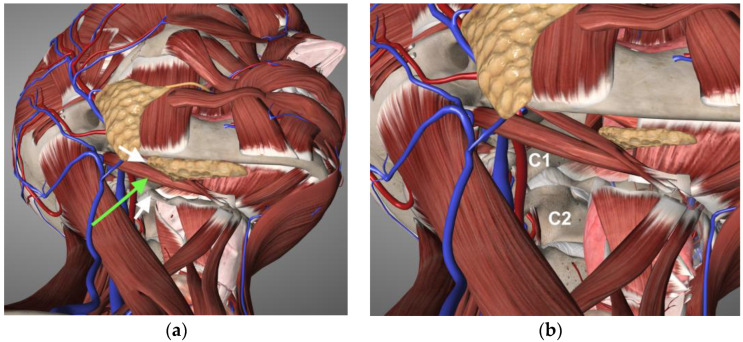
Three-dimensional anatomical renderings showing: (**a**) the landmarks for our approach (white arrows), i.e., the submandibular gland (superiorly) and the greater horn of the hyoid bone (inferiorly) and the direction of finger dissection (green arrow); (**b**) the obtained exposure of the CVJ, ranging from the lower aspect of C1 to the full extent of C2. The reconstructions were obtained with Essential Anatomy, copyright 3D4Medical, Elsevier.

The access to the prevertebral space in our approach is guided by two landmarks (Figure 2):

(1) Superiorly, the inferior belly of the submandibular gland, which is identified but not dissected or incised.
(2) Infero-medially, the greater horn of the hyoid bone, which represents the inferior starting point for the oblique dissection along the parapharyngeal and retropharyngeal spaces.

The surgical corridor is identified with finger dissection between these surgical landmarks in an ascending, lateral-to-medial direction. The retropharyngeal fibro-adipose tissue and the longus colli muscles are then dissected to expose the spine; the gained exposure is maintained with the use of Cloward’s self-retaining retractors. The lips of the retractors are inserted under the longus colli muscles bilaterally. No vessels or nerves must be identified or dissected, and no exposure or suspension of the submandibular gland is necessary. A neurosurgical microscope or an endoscope with 0° and 30° optics can be used to increase visualization.

## 3. Illustrative Cases

### 3.1. Case 1

**Figure 3:**
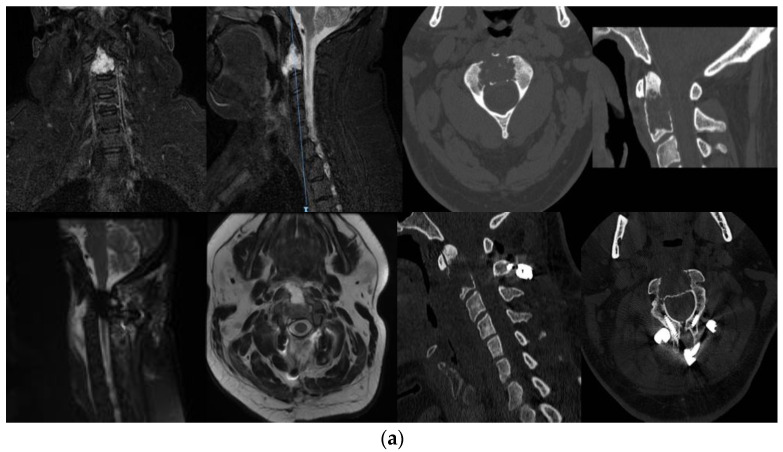

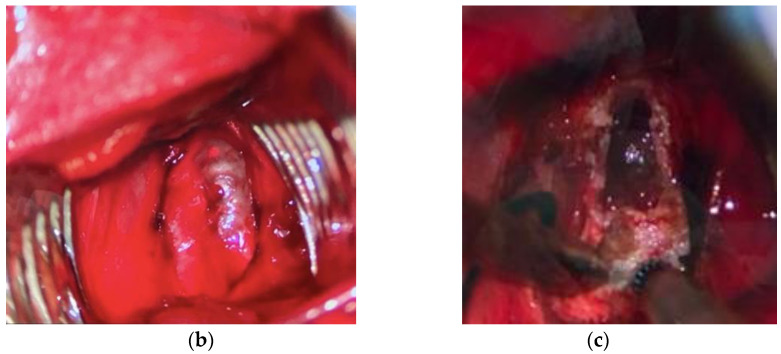
(**a**) Preoperative and post-operative CT and MRI images of patient 1, showing the T2-hyperintense lesion involving the body of C2; complete resection was achieved with subsequent C1–C2 posterior instrumentation with C1 lateral mass and C2 laminar screws. (**b**) Intraoperative image of the anterior aspect of the lesion, replacing the body of C2. (**c**) Intraoperative photograph showing the hollowed C2 body after resection of the lesion.

This was a thirty-nine-year-old man with an osteolytic somatic lesion of C2, identified with imaging studies performed for neck pain. MRI documented an osteolytic lesion of the axis with homogeneous contrast enhancement (Figure 3). No other disease locations were apparent on whole-body imaging. Neurological examination was negative.

After thorough discussion of the case, the patient underwent surgery in two steps. 

The first step involved removal of the lesion, which was accessed via a retropharyngeal approach as described above; we used intraoperative O-Arm navigation, which confirmed that the lesion could be entirely accessed. Gross total removal was obtained, leaving a shell of cortical odontoid bone. The second surgical step involved posterior C1–C2 instrumentation with C1 lateral mass screws and C2 laminar screws. Histopathology characterized the lesion as chordoma. 

The postoperative course was uneventful, and the patient was mobilized on postop day 1. Follow-up MRI and CT scans documented gross-total removal with correct positioning of the instrumentation (Figure 3). The patient therefore received proton beam therapy. At his 2-year follow-up, the patient was in good clinical and neurological condition.

### 3.2. Case 2

**Figure 4:**
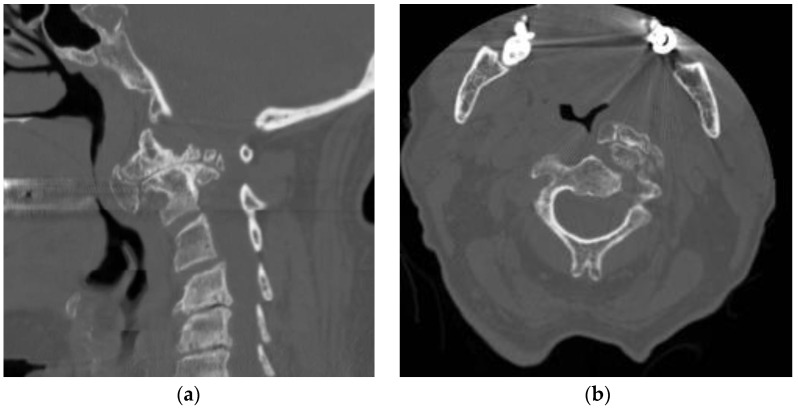

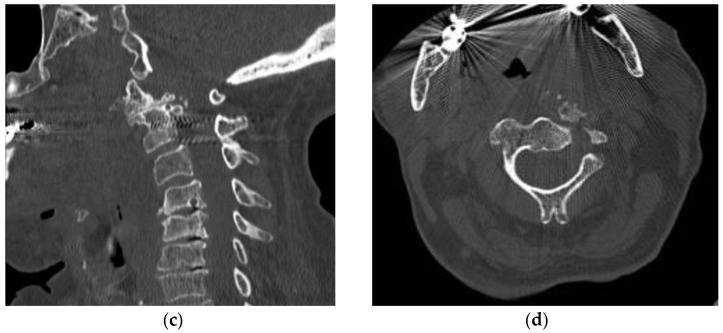
Preoperative (**a**,**b**) and postoperative (**c**,**d**) CT scans of patient 2, documenting a hyperostotic bone mass with three components involving C1 and the left C2 lateral mass. The component along the midline was resected obtaining a satisfactory reduction in the compression on the posterior pharyngeal wall.

This was an 81-year-old female patient with a history of progressive dysphonia over 2 years. Imaging documented a lesion with multiple exophytic degenerative bone products involving both C1 and C2 on the left (Figure 4). The patient’s neurological examination was significant for marked dysphonia and dysphagia. We discussed the case with the patient and her family, who agreed with the surgical indication.

Surgery was performed with O-Arm intraoperative navigation. The lesion was accessed via an ipsilateral (left) submandibular retropharyngeal approach as described above. Exposure of the lesion was sufficient but not optimal. However, due to the age of the patient and her comorbidities, we did not pursue a complete removal; instead, we debulked the lesion, obtaining a sufficient decompression (Figure 4). In the postoperative period, the patient complained of swallowing difficulty due to swelling of the pharyngeal wall, which improved rapidly. Laryngoscopic examination did not reveal any alteration in the swallowing mechanisms, aside from a mild impairment in tongue motility. Preoperative dysphonia progressively improved and completely recovered in two weeks. The patient was discharged home with no new neurological deficits besides the mild impairment in tongue motility. The patient died one year later due to unrelated causes.

### 3.3. Case 3

**Figure 5:**
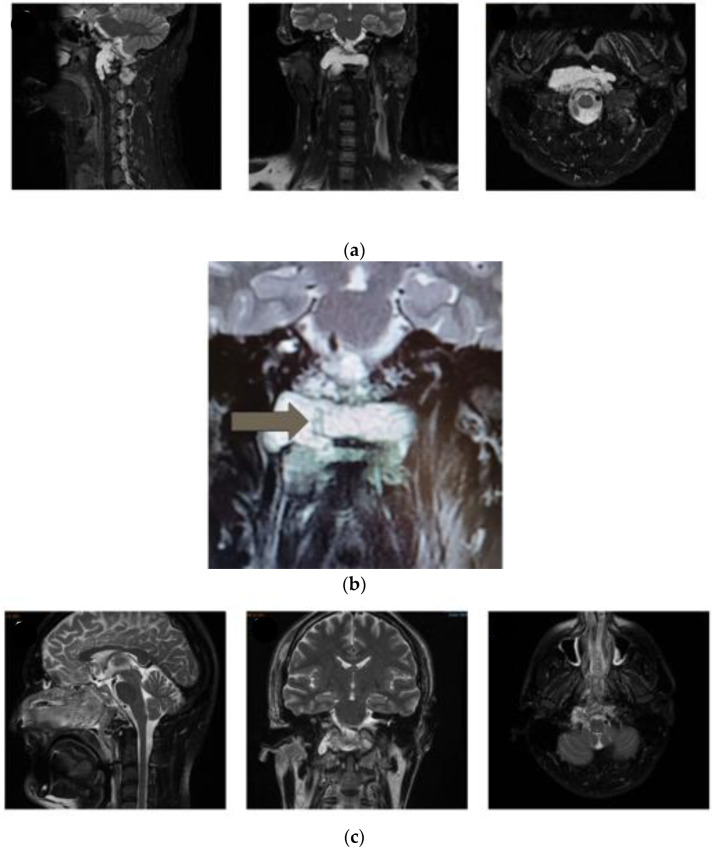
(**a**) Preoperative images of patient 3, showing the lesion involving the inferior clivus, right occipital condyle, and atlas (arch and right articular mass). (**b**) MRI obtained after the first unsuccessful attempt at resection via a submandibular approach, showing the biopsy tract (arrow). (**c**) Postoperative MRI after transoral–transnasal resection, showing near-total resection of the tumor with a small residue in the right lateral mass of the atlas (right image).

This case illustrates the limits of the submandibular retropharyngeal approach. The patient was a 44-year-old man with a two-year history of progressive headache and vertigo. Imaging studies showed an extradural, enhancing CVJ lesion infiltrating the inferior clivus, occipital condyles, and anterior arch and articular masses of the atlas (Figure 5). Neurological examination was normal. Since the patient had a history of Beçet disease (a rare condition that produces an autoimmune inflammation of the oral mucosa, resulting in multiple recurrent ulcers), both transnasal and transoral approaches were not considered as a first option. The lesion was then accessed via a submandibular retropharyngeal route. The exposure, however, appeared suboptimal, and we performed a biopsy. Histopathological examination was consistent with chordoma. Therefore, the patient underwent a second procedure in which removal of the lesion was obtained via a combined transnasal and transoral endoscopic route [7,9]. A small lateral residue infiltrating the articular masses of the atlas was left. The postoperative period was uneventful, and the patient was ambulatory on postoperative day one. No posterior fixation was performed due to refusal of the patient, who agreed to wear a rigid collar.

## 4. Discussion

### 4.1. History

The history of the submandibular retropharyngeal approach can be traced back to 1957, when Southwick and Robinson [10] described an extra-mucosal anterior approach (EMA) to the subaxial cervical region. In 1969, de Andrade and MacNab [10] developed a cranial variant of this approach, allowing for extension to the C1–C2 region. This technique was subsequently modified in 1987 by McAfee et al. [1], who added the resection of the submandibular (SM) gland and the transection of the intermediate tendon of the digastric muscle to allow for a wider exposure and better mobilization of the hypoglossal nerve. Other authors have described the usefulness of the submandibular retropharyngeal approach, providing wide exposure from the clivus to the upper cervical spine, as shown in Figure 1 [1,3,4,5,8,11].

Behari (et al.) presented his experience with this approach in five patients affected by high cervical extradural cord compression who underwent decompression and fusion following the traditional McAfee dissection steps. The authors reported some complications such as neurological deterioration in two patients and concluded that the approach is extremely useful and safe in accessing anteriorly situated lesions at the C1–C3 level, achieving a wide exposure and allowing for anterior fixation during the same surgical procedure [12].

Park et al. adopted the approach in 15 patients that underwent decompression and fusion on the upper cervical spine and concluded that the approach was feasible and without severe morbidities [13].

More recently, in 2010, Traynelis and Fontes [14] reported their experience with this approach, extending the exposure rostrally through careful soft tissue dissection and mobilization of the superior thyroid artery, without resecting the submandibular gland or transecting the digastric muscle.

A variant of the SMA was described by Ricciardi et al., who investigated on cadaveric specimens the feasibility of a simultaneous anterior decompression and fixation of the CVJ through a submandibular extramucosal route [15]. The authors concluded that this is feasible and could result in a shorter duration of surgery, avoiding complications related to both the transmucosal approach [16] and the prone position, although specific related risks exist. Ultimately, De Bonis [6] described a simplified submandibular approach in four steps, as compared to the more elaborate description by McAfee [1].

### 4.2. Surgical Procedure, Technical Nuances, and Potential Complications

In the SRA, the head is rotated about 30 degrees towards the contralateral side and extended. This allows for further cranial exposure, as the inferior rim of the mandible and mandibular angle naturally limit the access. According to the literature, the CVJ exposure and the working angles were comparable with those measured in transmucosal approaches (TMA), i.e., transoral and transnasal approaches (Figure 1) [12,13,16,17,18,19,20]. It has been previously reported that extramucosal approaches reduce the risk of dysphagia, dyslalia, snoring, and other postsurgical conditions related to the upper respiratory and oropharyngeal tracts [21]. However, as previously noted by several authors for the treatment of parapharyngeal tumors, the SRA is not free of complications, such as dysphagia and dysphonia due to due to hypoglossal nerve manipulation and/or damage to the superior laryngeal nerve, drooling due to damage to the inferior marginal mandibular branch of the facial nerve, and salivary fistula as a possible consequence of resection of the submandibular gland, which is advocated by some authors to increase exposure reaching up to the clivus [22]. Many authors suggest dividing the digastric and stylohyoid muscles to allow for greater mobilization of midline structures [11,21,23]; however, this has the potential for injuring the hypoglossal nerve, which runs close to the digastric belly. Other authors perform a complete dissection of this nerve to clearly identify and mobilize it [24,25,26,27]; nonetheless, nerve manipulation can lead to postoperative transient tongue weakness and thus speech and swallowing problems. Some authors underline the need to identify the thyrohyoid membrane together with the internal branch of SLN [24,25,26,28,29,30]; however, this step could carry the potential for nerve injury [13]. Transient palsy of the mandibular branch of the facial nerve has also been reported; some authors, therefore, advocate a lower skin incision, further from the mandible, to protect this nerve branch.

For the above-mentioned reasons, the McAfee approach represents a viable and strategic surgical approach, although complex and associated with potential significant comorbidities. To overcome these drawbacks, the group of De Bonis [6] proposed a simplification of the McAfee approach, with the rationale that the risks associated with the meticulous dissection involved in the latter approach were not justified by a significant increase in surgical exposure. The modified technique consists of four steps and involves dissection between the supero-lateral border of the hyoid bone and the digastric muscle tendon (Table 1).

This technique is illustrated in five surgical cases: two of them had a C2–C3 herniated disk with myelopathy, the other two had unstable Hangman fractures, whereas one had a C2–C3 osteophyte causing dysphagia. The authors explain how they were able to adequately access all these pathologies without the need to identify and dissect the bellies of the digastric muscle, hypoglossal nerve, etc., with no surgical complications.

### 4.3. Original Considerations and Simplification of the Approach

In our experience, dissection around the greater horn of the hyoid bone and digastric tendon is not necessary and could lead to inadvertent exposure and damage to the hypoglossal nerve. We have, therefore, developed a further simplification of the approach, comprising only three steps.

Our technique does not involve dissection of the tendon of the digastric muscle. For orientation, the greater horn of the hyoid bone is identified by palpation; this represents a crucial landmark, together with the inferior belly of the submandibular gland (see Section 2). We do not perform any dissection between the supero-lateral border of the hyoid bone and the digastric tendon, thus minimizing complications related to inadvertent damage to the hypoglossal nerve.

The main limitations of our technique are related to its simplicity. Since dissection of the anatomical structures is kept at a minimum, the working corridor, although adequate to access the antero-lateral portion of the CVJ, is not as comfortable as the one obtained with the conventional SRA. Our approach offers excellent access to the body of C2, the odontoid process, and the lower aspect of the anterior C1 arch, as illustrated in case 1 (midline C2 chordoma); it provides a sufficient access to paramedian pathology at the C1–C2 junction (case 2, paramedian C1–C2 hyperostosis), while the exposure of pathology that is located further cranially and laterally is suboptimal (case 3, C1 tumor with lateral extension). Of note, as stated above, the technique previously reported by De Bonis [6] was used to access pathology at the C2 and C3 levels.

To summarize, the optimal indication for our approach is represented by midline lesions involving C2 body and odontoid process, such as tumors, anterior compressive pathology (both of degenerative and malformative nature), and traumatic conditions.

For lesions that present an asymmetrical lateral extension, a contralateral approach (i.e., from the side opposite to the major extension of the lesion) is indicated, allowing us to reach up to the C1–C2 junction by working along the major axis of the pathology. For lesions located at the level of C1 or above, especially off the midline, the exposure provided by our approach is suboptimal. We therefore stress the importance of accurate patient selection to obtain the most out of this technique while benefiting from its simplicity and lower complication risk. 

**Figure 6:**
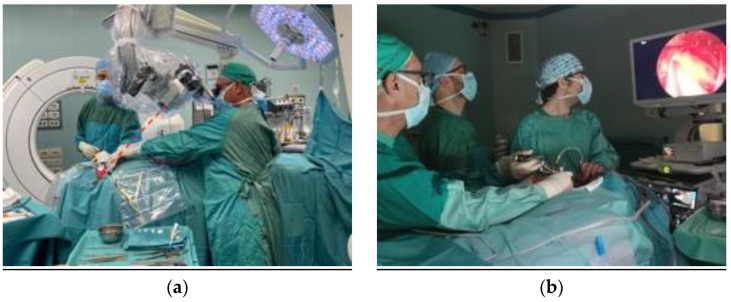
(**a**) Photograph obtained during the operation on patient 1, showing the visual and working angle. When targeting C2, it is possible to use a microscope. (**b**) For lesions located at C1 and above, endoscopic assistance is mandatory, as shown in this photograph obtained during surgery on patient 3.

From a technical perspective, a microscope can be use when working at the level of C2 (Figure 6); endoscopic assistance with 0° and 30° optics is instead mandatory when targeting C1. Even with the use of an endoscope, obtaining a proper access to C1 lesions can be difficult, especially when they are located off the midline.

## 5. Conclusions

The classic SRA is a demanding approach that requires detailed knowledge of submandibular region anatomy and ENT surgical support, and it carries a considerable risk of complications. Consequently, the SRA is rarely performed. In this paper, we propose a simplification of the traditional SRA, reducing the procedure to only three steps, allowing for more straightforward exposure of the antero-lateral CVJ.

Our technique is based on the identification of a natural anatomical corridor between two key landmarks, the inferior belly of the submandibular gland (superiorly) and the greater horn of the hyoid bone (infero-medially). No dissection is required besides opening the virtual space between the buccopharyngeal fascia and the carotid sheath. As shown through our case presentations, the oblique surgical route offers an optimal exposure of midline C2 pathology, with possible lateral and cranial extension to the contralateral C1–C2 junction. With endoscopic assistance, it is also possible to access C1 pathology, although with suboptimal results.

We have illustrated the similarities and differences between the classic Southwick and Robinson approach, its variations, and our modification of the SRA that makes it more easily suitable for CVJ pathology up to the C1–C2 junction, offering an alternative to the more commonly used anterior transmucosal approaches.

## Figures and Tables

**Table 1:** Description of the “standard” technique and its subsequent simplifications.

Mcafee et al. [1]	De Bonis et al. [6]	Our Technique
1. Positioning 2. Skin incision 3. Submandibular gland dissection 4. Facial artery ligation 5. Carotid artery identification and superior thyroid artery ligation 6. Digastric muscle transection 7. Digastric muscle belly dissection 8. Hypoglossal nerve dissection and elevation 9. Hyoid bone lateral horn dissection 10. Superior laryngeal nerve dissection and retraction 11. Retropharyngeal space exposure	1. Positioning 2. Incision of the superficial layers 3. Dissection of the plane between greater horn of the hyoid bone and the tendon of the digastric muscle supero-laterally 4. Retropharyngeal space exposure	1. Positioning 2. Incision of the superficial layers 3. Blunt dissection of the parapharyngeal space, using the greater horn of the hyoid bone and the submandibular gland as landmarks that are palpated but not dissected

## Data Availability

All the data generated in the present study have been included in this paper after anonymization.
